# Single-step route to diamond-nanotube composite

**DOI:** 10.1186/1556-276X-7-535

**Published:** 2012-09-27

**Authors:** Deepak Varshney, Majid Ahmadi, Maxime J-F Guinel, Brad R Weiner, Gerardo Morell

**Affiliations:** 1 Department of Physics, University of Puerto Rico, San Juan, PR, 00936-8377, USA; 2 Institute of Functional Nanomaterials, University of Puerto Rico, San Juan, PR, 00931-3334, USA; 3 Department of Chemistry, University of Puerto Rico, San Juan, PR, 00936-8377, USA

**Keywords:** Carbon nanotubes, Diamond crystals, Chemical vapor deposition

## Abstract

Candle wax was used as a precursor for the production of a diamond-nanotube composite in a single step. The composite films were fabricated by sulfur-assisted hot-filament chemical vapor deposition technique. The morphology of the composite films was analyzed by scanning electron microscopy and transmission electron microscopy. Raman spectra of the films show characteristic diamond band at 1,332 cm^−1^, D-band around 1,342 cm^−1^, and graphitic G-band around 1,582 cm^−1^. The electron energy-loss spectroscopy recorded at the carbon K-edge region shows signature features of diamond and carbon nanotube in the fabricated material. The ability to synthesize diamond-nanotube composites at relatively low temperatures by a single-step process opens up new possibilities for the fabrication of nanoelectronic devices.

## Background

Since the documented discovery of carbon nanotubes (CNTs) in 1991 by Iijima [1] and the realization of their unique physical properties, including mechanical, thermal, and electrical, many investigators have endeavored to fabricate advanced CNT composite materials that exhibit one or more of these properties. Integration of CNTs with various kinds of materials, leading to the fabrication of composites possessing properties of the individual components with a synergistic effect, has gained growing interest. Carbon composites, such as diamond-nanotube, have various industrial applications because of their outstanding physical and chemical properties. CNTs are tiny rolled-up tubes of *sp*^2^-hybridized carbon that possess unique electronic transport properties and are the strongest known material, while diamond, consisting of *sp*^3^-hybridized carbon, exhibits high hardness, stable surface chemistry, and good biocompatibility [2,3]. The exceptional mechanical and electronic properties of CNTs and diamond make them promising candidates for use in microelectronic devices. A diamond-nanotube composite has excellent thermal conductivity and field emission characteristics and finds applications in various fields that require a combination of good thermal and electrical properties such as wear-resistant coatings, thermal management of integrated circuits, field emission devices, and electrical field shielding in MEMS and microelectronics [4].

There are several recent reports on the development of hybrid materials comprising of diamond and CNTs [4-6]. However, the reported methods have either two distinct seeding sources or a tedious two-step fabrication route which requires the use of a catalyst to provide initial nucleation sites for growth of both CNTs and diamond. To enhance the nucleation rate during diamond film growth, various techniques such as ultrasonic seeding [7], surface scratching, and bias-enhanced nucleation have been applied [8,9]. All the above techniques are mechanically abrasive and adversely affect the use of substrate for electronic applications, thus fueling the need for precursors that are cheap and handy and do not harm the substrate surface. Also, the transition metal catalysts, *viz.* Fe, Co, and Ni, that are used for CNT growth [10-12] react unfavorably with materials found in circuits and composites. Moreover, catalyst nanopowder can be toxic and cause problems in clean room environments. The present paper reports the catalyst-free growth of CNTs that is seeded by paraffin wax.

There are many reports on the fabrication of single-walled carbon nanotubes from polymers using metal catalyst [13,14]. In all previously reported techniques, the hybrid material was fabricated in two separate steps, each for metal-catalyzed CNT growth and diamond nanoparticle seeded diamond growth, but a single-step fabrication of diamond-nanotube composite using a single seeding source has been difficult. Here, we report the fabrication of diamond-nanotube composite by hot-filament chemical vapor deposition (HFCVD) in a single-step process, where paraffin wax is utilized as a seeding material for both diamond and carbon nanotubes. In the present report, trace amounts of sulfur have been used in the fabrication process to aid the formation of CNTs. The role of sulfur is explained in the ‘Results and discussion’ section. This paper demonstrates a new synthetic pathway for diamond-nanotube composite utilizing candle wax (paraffin wax), a cheap and widely used hydrocarbon.

## Methods

Polycrystalline copper substrates (99.9% pure, 0.5-mm thick, and 14-mm disk diameter) were hand polished with 600-grit sandpaper on both sides to make them flat. One side was further polished with 1,000-, 1,500-, and 2,000-grit sandpaper to smoothen the surface. The substrates were then cleaned in an ultrasonic bath with 2-propanol for 15 min and then dried with nitrogen. About 5 to 10 gm of candle wax (commercially available) was melted on a hot plate in a glass beaker by heating it to a temperature of 120°C at a rate of 15°C/min. A small portion of this melt was transferred onto a copper disk substrate with a thickness of 500 to 700 nm and allowed to cool to room temperature. The disks were then placed in the HFCVD chamber and exposed to a gas mixture of 0.3% methane and 99.7% of hydrogen (consisting of 500 ppm of H_2_S) at a constant pressure of 20 Torr and a total gas flow of 100 sccm. The reaction was activated for 3 h by a rhenium filament (8 cm in length and 0.5 mm in diameter) positioned 8 mm above the substrate. The temperature of the substrate and the filament was at approximately 550°C and 2,500°C, respectively.

The Raman scattering spectra were obtained using a triple monochromator (ISAJ-Y Model T 64000, HORIBA Ltd., Kyoto, Japan) with around 1 cm^−1^ resolution using the 514.5-nm line of Ar laser. The morphologies of the as-deposited materials were determined using a JEOL JEM-7500 F scanning electron microscope (SEM; JEOL Ltd., Tokyo, Japan).

The samples were also analyzed using a Carl Zeiss LEO 922 energy-filtered transmission electron microscope (TEM; Carl Zeiss AG, Oberkochen, Germany) operated at 200 kV, including an omega-type energy filter to study the electron energy-loss spectroscopy (EELS). The TEM samples were prepared by immersing an ultrathin carbon-coated copper grid into an ultrasonicated suspension of the fabricated material in ethanol. The Fourier transform infrared spectroscopy (FTIR) was carried out using a Bruker Tensor 27 instrument (Bruker Optik GmbH, Ettlingen, Germany). The samples were prepared by melting 0.5 g of wax onto the substrate and allowing it to cool to room temperature.

## Results and discussion

Candle wax is generally composed of paraffin, which is made of heavy straight-chain hydrocarbons obtained from crude petroleum oil [15]. Crystalline paraffin waxes are solid and crystalline mixtures of hydrocarbons consisting of linear n-alkane and branched iso- and cyclo-alkanes with carbon lengths ranging from C_16_ to C_30_ and higher [16,17]. The paraffin crystallites seem to act as nucleation sites for diamond and carbon nanotube growth in the presence of hydrocarbon radicals and atomic hydrogen in the chemical vapor deposition (CVD) system, resulting in microcrystalline diamond-carbon nanotube films [18].

**Figure 1:**
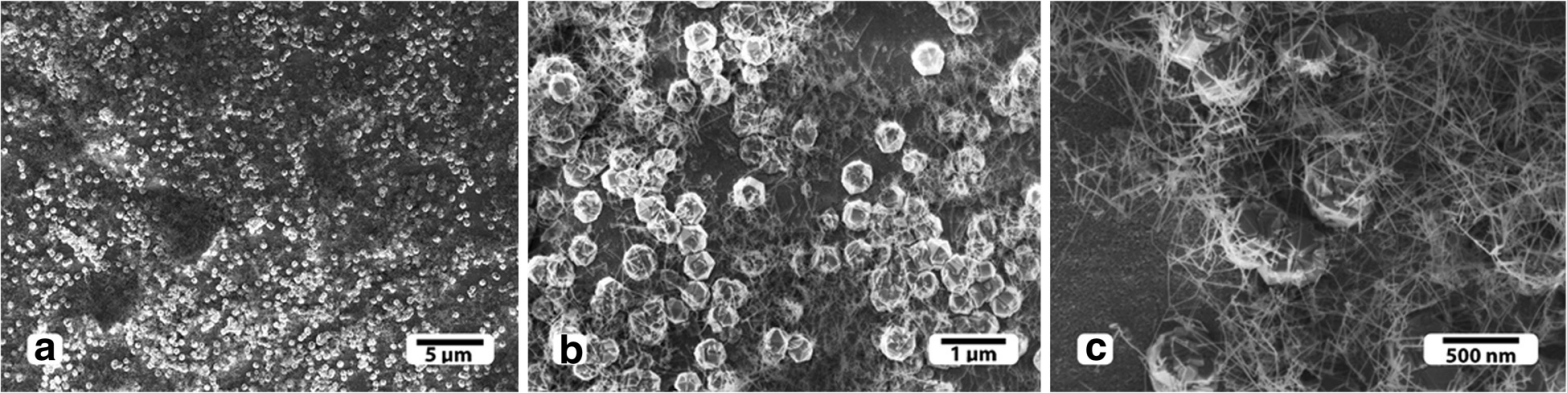
**SEM images of thediamond-nanotube composite film. **(**a**) and (**b**) show diamond microcrystals entangled in a carbon nanotube network. (**c**) shows the diamond microcrystals with size ranging from 0.4 to 1.0 μm.

The morphology of the diamond-nanotube composite films was ascertained by SEM. Figure 1a,b,c reveals a network of carbon nanotubes entangling the diamond microcrystals. The microcrystals with size ranging from 0.4 to 1.0 μm are randomly nucleated in a web of several-microns-long carbon nanotubes. From the SEM images (Figure 1a,b,c), we notice that the distribution of the diamond microcrystals and the nanotubes on the substrate has a bimodal distribution, which implies that the diamond crystals and nanotubes have been fabricated in a single step.

**Figure 2:**
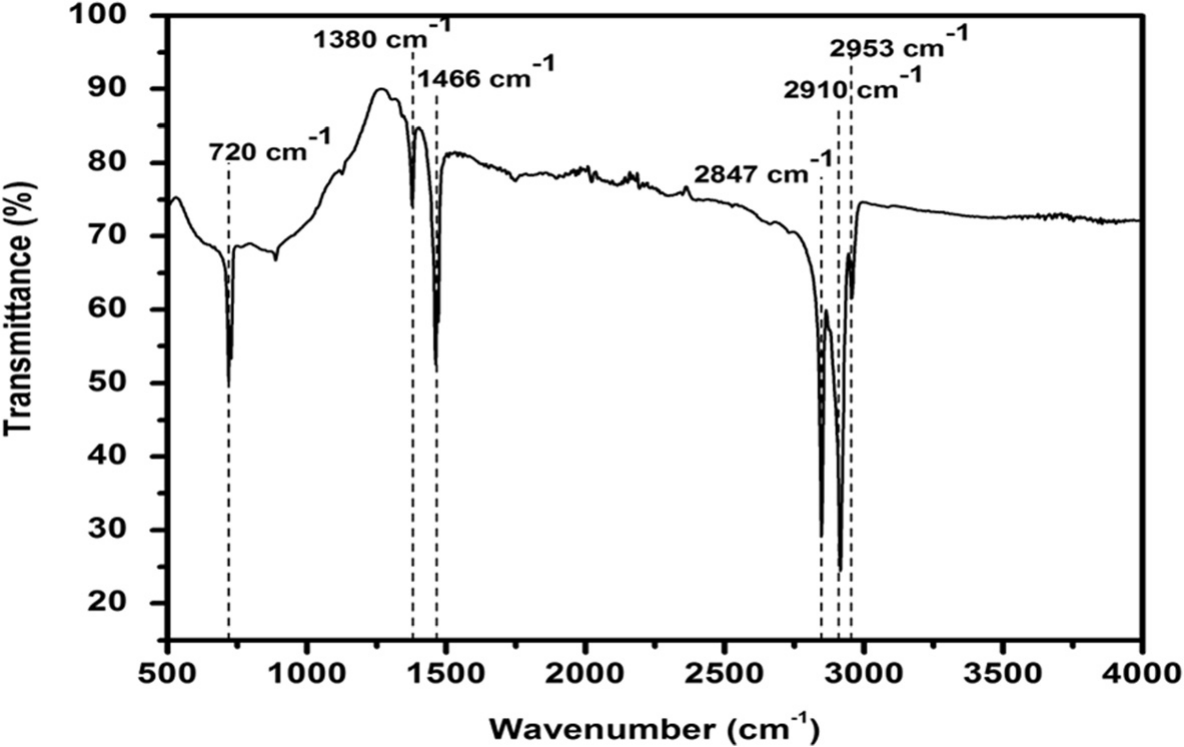
**FTIR spectrum of theparaffin wax melt.** It confirms the absence of oxygen in the paraffin melt.

FTIR spectroscopy was used to analyze the chemical composition of paraffin wax, and the obtained spectrum is shown in Figure 2, which reveals the presence of carbon-hydrogen stretching and bending absorption bands in the range of 1,000 to 3,000 cm^−1^. The symmetric carbon-hydrogen bending absorption of the CH_3_ group at 1,380 cm^−1^, the CH deformation around 1,460 cm^−1^, and the CH_2_ rocking absorption band at 725 cm^−1^ confirm the linear saturated aliphatic structure of the paraffin wax [19]. The FTIR spectrum of the paraffin melt does not contain any bands for oxygen, confirming the absence of oxygen in the paraffin melt.

**Figure 3:**
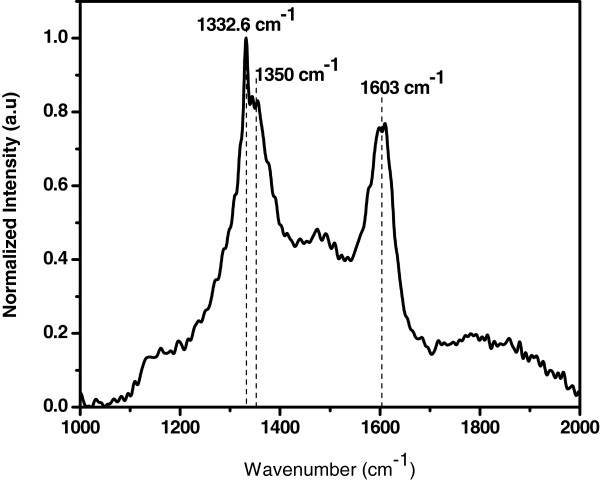
**Raman spectrum of thediamond-nanotube composite film.** The spectrum shows bands corresponding to the presence of diamond and carbon nanotubes in the composite film.

The Raman spectra of the wax-coated substrate (Figure 3) show the typical vibrational modes of paraffin, which is the most common polymer in industrial and everyday household use. The characteristic fundamental vibrational modes observed between 1,000 and 2,000 cm^−1^ are frequently used to study morphological structure. The spectra show distinctive Raman contributions at 1,063, 1,133, 1,171, 1,296, 1,418, 1,441, and 1,463 cm^−1^ that are attributed to C-C (carbon-carbon) stretching and the CH_2_ and CH_3_ deformations [20] which arise from the straight-chain hydrocarbon structure of the seeding polymer [21]. Figure 3 shows the Raman spectrum of the composite film revealing a sharp and intense peak at 1,332.6 cm^−1^, which is the signature of *sp*^3^-bonded C in diamond phase [22]. The shoulder around 1,350 cm^−1^ corresponds to the disorder induced in the *sp*^2^ carbon [23]. The band at about 1,603 cm^−1^ corresponds to *sp*^2^-hybridized carbon, indicating the presence of graphitic carbon [24]. The absence of typical vibrational modes of candle wax confirms that it acts as a seeding source for diamond and also for CNTs. We propose a phenomenological explanation of the synthesis of diamond-nanotube composite from a single seeding source (candle wax).

**Figure 4:**
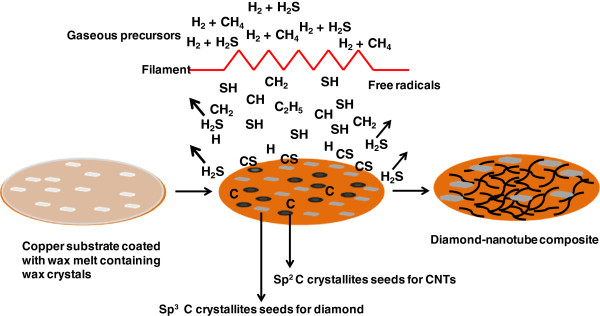
**Schematic representation of themechanism of diamond-nanotube compositefilm formation.** Formation of diamond-nanotube composite having diamond crystals (gray in color) entangled in the carbon nanotubes (black lines).

The genesis of diamond-nanotube composite from candle wax seeds can be understood qualitatively through a mechanism (Figure 4) based on the current understanding of the CVD of diamond and CNTs and the decomposition of candle wax. Candle wax consists of long chains of n-alkanes with melting point in the range of 58°C to 62°C and a flash point starting at 204°C. Under HFCVD conditions above the flash point, the candle wax decomposes, leaving behind *sp*^2^-C and *sp*^3^-C crystallites [22,25] on the Cu substrate surface, which act as nuclei for the CNTs and diamond, respectively. Another factor that influences the crystal formation is the interactive forces on the surface [26]. Any non-carbide-forming substrate, such as Cu, Ag, or Au, provides a surface with minimal surface interactions, thus facilitating the formation of *sp*^2^-C and *sp*^3^-C crystallites. Under typical HFCVD conditions, atomic H etches away the *sp*^2^-C crystallites, thus favoring the formation of microcrystalline diamond. However, the addition of trace amounts of sulfur, in the form of hydrogen sulfide, to the chemical vapor deposition reaction leads to profound changes in the gas phase chemistry and the surface chemistry. The high temperature around the filament leads to the dissociation of H_2_S and CH_4_ to form SH radicals along with carbon radicals. When the radicals move away from the filament to a cooler region at the substrate surface, the active SH radicals combine with C radicals to form CS radicals, which combine with hydrogen atoms to form H_2_S again while depositing carbon atoms onto the substrate surface [27]. The net effect is analogous to hydrogen depletion, thus avoiding the preferential etching of *sp*^2^-C crystallites. These conditions promote the growth of CNTs by surface diffusion of carbon, leading to a CNT growth mechanism known as the ‘vapor-solid surface-solid’ mechanism [28]. Simultaneously, the *sp*^3^-C crystallites grow into diamond microcrystals through the usual mechanism. In essence, the presence of sulfur increases the CNT growth rate, making it more rapid than the etching rate of CNTs in the presence of atomic hydrogen. These results open new possibilities for diamond and carbon nanotubes to be grown simultaneously using a cheap seeding source without the use of a metal catalyst.

**Figure 5:**
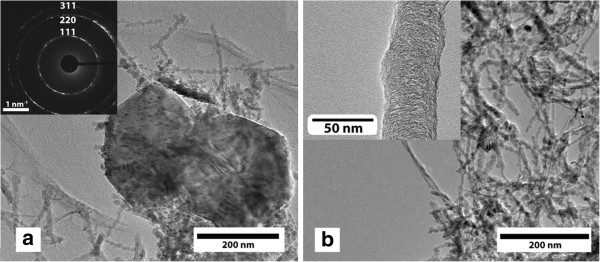
**TEM images of thediamond-nanotube composite film. ** (**a**,**b**) TEM image showing the diamond microcrystals present in the diamond-nanotube composite film synthesized using paraffin wax. The inset of Figure 5a shows the corresponding electron diffraction pattern indexed to diamond with its {111}, {220} and {311} reflections. The inset of Figure 5b shows the magnified image of the carbon nanotubes.

TEM studies were used for further characterization of the diamond-nanotube composite films. Figure 5 shows the TEM images of the carbon nanotubes and diamond particles. Figure 5a shows diamond crystals with size ranging from 350 to 450 nm together with carbon nanotubes. The inset of Figure 5a shows selected area electron diffraction pattern recorded from the microcrystalline diamond. The polycrystalline diffraction rings correspond to {111}, {220}, and {311} and are in agreement with the diamond lattice structure. Figure 5b shows the TEM image of the composite film revealing the presence of carbon nanotubes. The inset of Figure 5b shows the magnified image of carbon nanotubes, which were found to have a diameter in the range of 40 to 50 nm.

**Figure 6:**
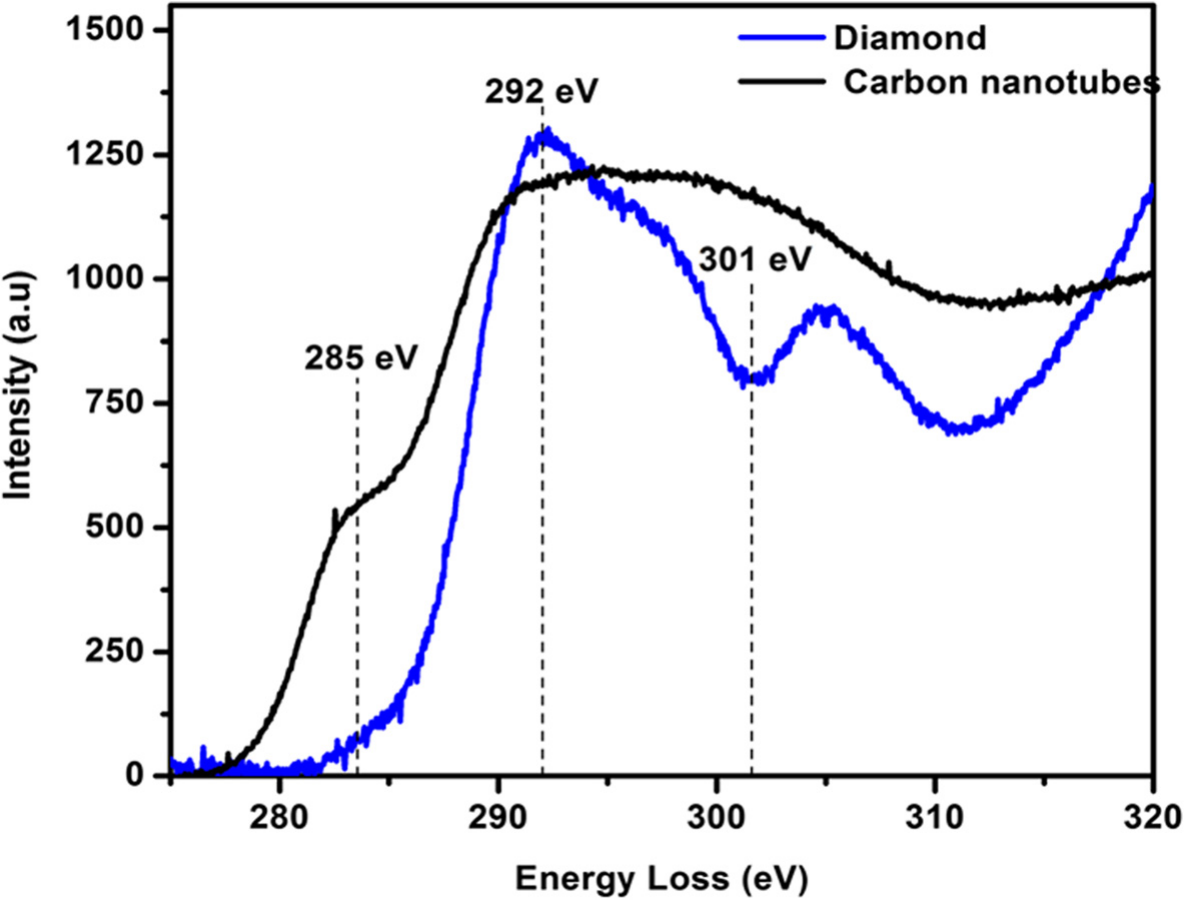
**EELS spectra of thediamond-nanotube composite film.** The obtained spectra confirm the presence of carbon nanotubes and diamond microcrystals.

EELS were recorded in the carbon K-edge region near-edge structure between 285 to 310 eV and was used to investigate the structure of the composite film (shown in Figure 6). EELS were recorded on different regions of the composite film, which are shown in Figure 5a and are indicated by arrows. The EELS spectrum of the tubular structure (black line) shows edge around 285 eV due to 1*s* to low-lying antibonding π* transitions and around 292 eV due to the 1*s* to σ* transition. The energy-loss spectrum recorded from the diamond particles (blue line) clearly shows the presence of a dip around 301 eV due to the second absolute bandgap of diamond [29], which distinguishes it from carbon nanotubes.

## Conclusions

A simple and reproducible single-step method for the fabrication of diamond-nanotube composite films was reported. The characteristic features of both carbon nanotubes and diamond in the composite film were evident from the Raman spectroscopy, EELS, SEM, and TEM results. To our knowledge, this is the first example of a sulfur-based growth of diamond-nanotube composite. The low-temperature fabrication of the present diamond-nanotube composite without the use of a metal catalyst and in a single-step process opens up new possibilities for the fabrication of composite-based nanoelectronic devices.

## Competing interests

The authors declare that they have no competing interests.

## Authors’ contributions

DV synthesized and characterized the hybrid films and wrote the manuscript. MA and MJFG did the SEM and TEM analyses. BRW and GM supervised the study. All authors read and approved the final manuscript.

## Authors’ information

DV and MA are PhD students at the University of Puerto Rico (UPR). MJFG is an assistant professor at the Department of Physics and Department of Chemistry at UPR. BRW is a dean and professor at UPR. GM is a professor and the director of the Department of Physics at UPR.
